# Trimethylamine N-Oxide Improves Exercise Performance by Reducing Oxidative Stress through Activation of the Nrf2 Signaling Pathway

**DOI:** 10.3390/molecules29040759

**Published:** 2024-02-06

**Authors:** Hong Zou, Yu Zhou, Lijing Gong, Caihua Huang, Xi Liu, Ruohan Lu, Jingjing Yu, Zhenxing Kong, Yimin Zhang, Donghai Lin

**Affiliations:** 1 Key Laboratory for Chemical Biology of Fujian Province, MOE Key Laboratory of Spectrochemical Analysis & Instrumentation, College of Chemistry and Chemical Engineering, Xiamen University, Xiamen 361005, Chinalancyliu2584@163.com (X.L.); lrh626718@163.com (R.L.); 2 Physical Education Department, Xiamen University, Xiamen 361005, China; zou_hong0402@126.com; 3 Key Laboratory of Ministry of Education of Exercise and Physical Fitness, Beijing Sport University, Beijing 100084, China; lijing.gong@bsu.edu.cn (L.G.); yujingjing@bsu.edu.cn (J.Y.); kongzhenxing@bsu.edu.cn (Z.K.); 4 China Institute of Sports and Health, Beijing Sport University, Beijing 100084, China; 5 Research and Communication Center of Exercise and Health, Xiamen University of Technology, Xiamen 361021, China; caihua.huang@foxmail.com; 6 Affiliated High School of Minnan, Normal University, Zhangzhou 363005, China

**Keywords:** TMAO, exercise performance, oxidative stress, Nrf2 signaling pathway

## Abstract

Trimethylamine N-oxide (TMAO) has attracted interest because of its association with cardiovascular disease and diabetes, and evidence for the beneficial effects of TMAO is accumulating. This study investigates the role of TMAO in improving exercise performance and elucidates the underlying molecular mechanisms. Using C2C12 cells, we established an oxidative stress model and administered TMAO treatment. Our results indicate that TMAO significantly protects myoblasts from oxidative stress-induced damage by increasing the expression of Nrf2, heme oxygenase-1 (HO-1), NAD(P)H dehydrogenase (NQO1), and catalase (CAT). In particular, suppression of Nrf2 resulted in a loss of the protective effects of TMAO and a significant decrease in the expression levels of Nrf2, HO-1, and NQO1. In addition, we evaluated the effects of TMAO in an exhaustive swimming test in mice. TMAO treatment significantly prolonged swimming endurance, increased glutathione and taurine levels, enhanced glutathione peroxidase activity, and increased the expression of Nrf2 and its downstream antioxidant genes, including HO-1, NQO1, and CAT, in skeletal muscle. These findings underscore the potential of TMAO to counteract exercise-induced oxidative stress. This research provides new insights into the ability of TMAO to alleviate exercise-induced oxidative stress via the Nrf2 signaling pathway, providing a valuable framework for the development of sports nutrition supplements aimed at mitigating oxidative stress.

## 1. Introduction

Trimethylamine N-oxide (TMAO, the chemical structure is shown in Figure S1) has attracted interest because of its association with cardiovascular disease [1] and type 2 diabetes [2,3]. Elevated levels of TMAO have been suggested as a potential biomarker for the early detection of metabolic syndrome [4], although recent evidence suggests that TMAO may be a consequence of the disease rather than a causative factor [5]. Evidence for the beneficial effects of TMAO is accumulating. In particular, TMAO has shown protective properties for INS-1 beta cells and rat pancreatic islets under conditions of diabetic glucolipotoxicity [6]. In addition, studies have highlighted TMAO’s ability to protect MC65 cells, decrease markers of oxidative damage, and mitigate oxidative stress in the endoplasmic reticulum of streptozotocin-induced diabetic rats [7,8]. Our previous research [9] has demonstrated the efficacy of TMAO in reducing antioxidant stress in H_2_O_2_-induced C2C12 cells. However, the specific mechanisms by which TMAO counteracts oxidative stress remain to be elucidated.

The transcription factor nuclear factor erythroid 2-related factor 2 (Nrf2), a member of the basic leucine zipper (bZIP) protein family, serves as a central orchestrator of the cellular antioxidant defense mechanism, directing the expression of over 200 cytoprotective genes in the wake of oxidative stress [10,11]. Under normal conditions, Nrf2 activity is modulated by Kelch-like ECH-associated protein 1 (Keap1) [12,13] to maintain homeostasis. However, under conditions of oxidative stress, Nrf2 undergoes nuclear translocation [14,15,16] and triggers the transcription of key antioxidant genes, including heme oxygenase-1 (HO-1) [17], NAD(P)H dehydrogenase (quinone 1) (NQO1) [18], and catalase (CAT) [19].

The beneficial effects of habitual exercise have been widely recognized and documented [20,21]. However, high-intensity exercise significantly increases the production of reactive oxygen species (ROS), which temporarily disrupts the balance between ROS generation and neutralization. Although exercise-induced ROS play a pivotal role in improving muscle performance, their excessive proliferation may disrupt the delicate balance between pro- and antioxidants within cellular structures, culminating in oxidative stress and subsequent impairment of muscle contractility [22,23]. Numerous studies have demonstrated that both prolonged and intense physical activity induce oxidative stress in humans [24], and that this stress serves as a significant marker of muscle fatigue [21]. Enzymatic antioxidants, including catalase (CAT), glutathione peroxidase (GSH-Px), and glutathione, play a critical role in counteracting exercise-induced oxidative stress, thereby reducing the likelihood of cellular damage during physical exertion [23,25,26]. High-intensity exercise can deplete these endogenous antioxidants; however, replenishment through exogenous antioxidant supplementation has shown promise in preventing their reduction, leading to increased interest in antioxidant supplementation as a strategy to combat exercise-induced oxidative stress [27].

In this study, we aimed to elucidate the molecular mechanisms underlying the effects of TMAO and explore its potential application in enhancing exercise performance in mammals. We showed that TMAO treatment significantly enhanced the proliferative capacity of C2C12 myoblasts and significantly ameliorated oxidative stress-induced damage. This was achieved through the upregulation of Nrf2 and its downstream genes, coupled with the activation of the Nrf2 signaling pathway. In addition, our results clearly showed that TMAO supplementation significantly prolonged the exhaustive swimming time of mice. This study makes a notable contribution to the field by advancing our understanding of TMAO as a sports nutritional supplement with the potential to improve muscle performance and reduce exercise-induced oxidative stress.

## 2. Results

### 2.1. TMAO Prevented H_2_O_2_-Induced Damage in C2C12 Cells

**Figure 1:**
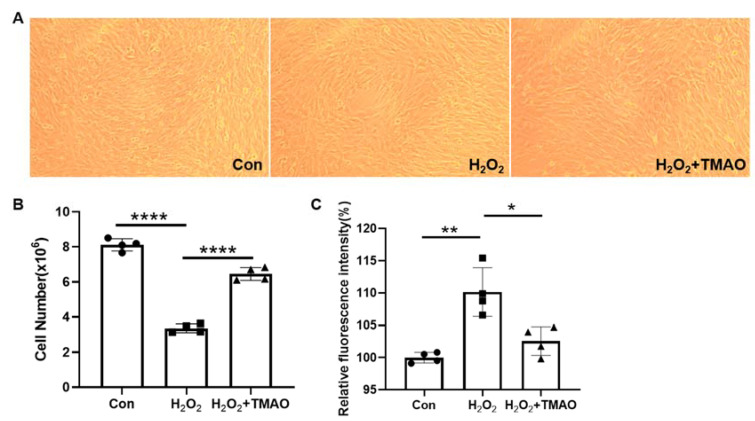
TMAO supplementation apparently improved the proliferation ability of H_2_O_2_-impaired C2C12 cells. (**A**) Representative morphological images of the cells. (**B**) Statistical analysis of cell number by automated cell counting. (**C**) Intracellular ROS levels measured by fluorescence intensity. Round: Con, Square: H_2_O_2_, Up Triangle: H_2_O_2_ + TMAO. Statistical significance: * *p* < 0.05; ** *p* < 0.01; **** *p* < 0.0001.

In our previous research [9], we found that TMAO can alleviate oxidative stress and identified the metabolic changes in C2C12 cells induced by H_2_O_2_ administration and TMAO supplementation. To further explore the mechanisms by which TMAO reduces oxidative stress, in this study we developed an oxidative stress model of C2C12 cells using 0.5 mM H_2_O_2_ and evaluated the effect of 5 mM TMAO supplementation on oxidative stress-related damage, consistent with the methodology of previous studies (Figure S2). Our results showed a significant decrease in both cell number and proliferative capacity following H_2_O_2_ treatment (*p* < 0.0001) (Figure 1A,B). Notably, TMAO supplementation resulted in an increase in cell number and density as shown in the morphological images. In addition, we measured intracellular ROS levels in the three groups of C2C12 myoblasts with a H_2_DCFDA probe. Significantly, TMAO supplementation eliminated the accumulated intracellular ROS induced by H_2_O_2_ (Figure 1C). This suggests that TMAO supplementation can potentially improve the proliferation ability of H_2_O_2_-impaired C2C12 cells.

### 2.2. TMAO Promoted Expression Levels of Nrf2, HO-1, NQO1, and CAT in H_2_O_2_-Impaired C2C12 Cells

**Figure 2:**
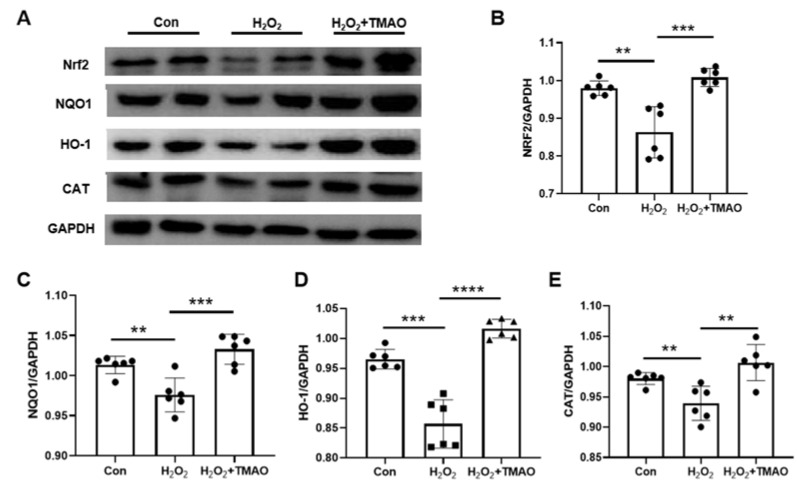
TMAO supplementation markedly promoted the expression of Nrf2 and its downstream genes in H_2_O_2_-impaired C2C12 cells. (**A**) Expression of NQO1, HO-1, Nrf2, and CAT analyzed by Western blotting. (**B**–**E**) Expression levels of Nrf2, NQO1, HO-1, and CAT. Round, Con; Square, H_2_O_2_; Up Triangle, H_2_O_2_ + TMAO. Statistical significance: ** *p* < 0.01; *** *p* < 0.001; **** *p* < 0.0001.

Nrf2 is a key regulator of cellular responses to external stressors and plays a critical role in coordinating antioxidant defense mechanisms [13]. The Keap1-Nrf2-ARE pathway has been recognized as a primary regulatory system in the management of antioxidant stress [15]. Extensive research has highlighted the ability of Nrf2 to increase the expression of its downstream antioxidant genes, including HO-1, NQO1, CAT, and glutathione, thereby providing essential antioxidant protection [28,29,30]. Our results (Figure 2) highlight a significant decrease in the expression levels of Nrf2, NQO1, HO-1, and CAT after administration of 0.5 mM H_2_O_2_. In contrast, supplementation with TMAO significantly restored these levels, suggesting its efficacy in counteracting the H_2_O_2_-induced reduction in the expression of these key proteins integral to antioxidant defense.

### 2.3. Knockdown of Nrf2 Decreased the Expression of Nrf2 and Its Downstream Genes in C2C12 Cells

To confirm the critical role of Nrf2 in the antioxidant function of TMAO, we used a commercial Nrf2 knockdown kit, following the instructions provided. Optimal concentrations of siRNA Nrf2 and the transfection agent Lip2000 were determined, and 40 nM siRNA Nrf2 and 32 nM Lip2000 were used for the cell experiment.

**Figure 3:**
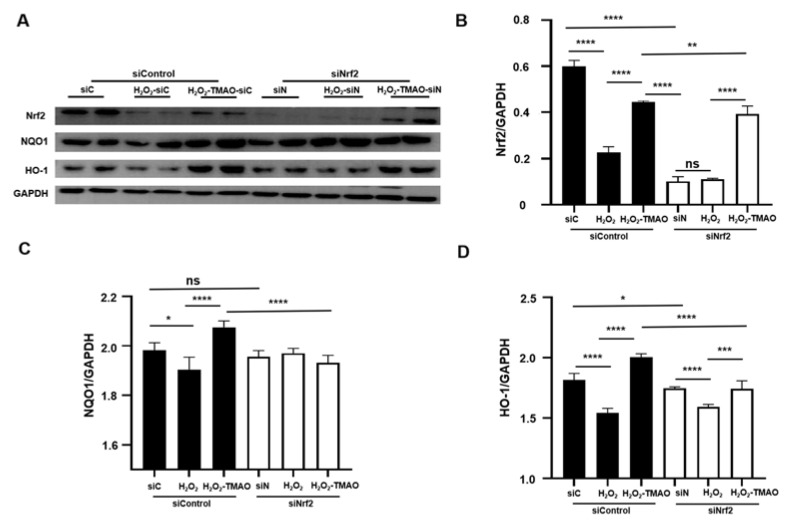
Knockdown of Nrf2 by siRNA significantly decreased the expression levels of Nrf2 and its downstream genes. (**A**) Western blot of Nrf2, NQO1, and HO-1. (**B**–**D**) Expression levels of Nrf2, NQO1, and HO-1. Statistical significance: ns *p* > 0.05, * *p* < 0.05; ** *p* < 0.01; *** *p* < 0.001, **** *p* < 0.0001.

Compared to untreated control siRNA (siC) cells, untreated siRNA Nrf2 (siN) cells showed significantly lower expression levels of Nrf2 and its downstream genes, with Nrf2 showing the most pronounced decrease (Figure 3). The H_2_O_2_-treated siC cells showed significantly reduced expression levels of Nrf2, NQO1, and HO-1 compared to untreated siC cells. However, H_2_O_2_-treated siN cells did not show significant changes in the expression levels of Nrf2, NQO1, and HO-1 compared to untreated siN cells.

Strikingly, TMAO treatment effectively reversed the reduced expression levels of these proteins in both H_2_O_2_-TMAO-treated siN and siC cells, regardless of Nrf2 knockdown (Figure 3). In addition, the expression levels of Nrf2 and HO-1 were lower in siN cells compared to siC cells. The expression levels in H_2_O_2_-TMAO-treated siN cells were significantly lower than those in H_2_O_2_-TMAO treated siC cells. Notably, the fold increase in NQO1 and HO-1 expression in H_2_O_2_-TMAO-treated siC cells was significantly greater than that in H_2_O_2_-TMAO-treated siN cells. These results suggest that TMAO treatment alleviates oxidative stress-induced damage by increasing the expression of Nrf2 and its downstream antioxidant genes.

### 2.4. TMAO Increased the Exhaustive Swimming Times of Mice

**Figure 4:**
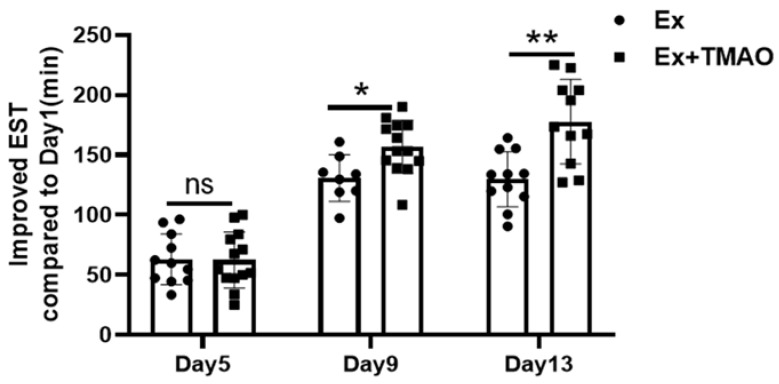
TMAO treatment significantly increased the exhaustive swimming times of mice on Days 9 and 13 compared to Day 1 of treatment. Statistical significance: ns, *p* > 0.05; * *p* < 0.05; ** *p* < 0.01.

To assess the efficacy of TMAO in improving exercise performance, we measured exhaustive swim time (EST) in both the exercise (Ex) and TMAO-treated exercise (Ex + TMAO) groups of mice on Days 1, 5, 9, and 13 following treatment. Compared to the Ex mice, the Ex + TMAO mice showed a gradual and significant increase in EST over the course of treatment, particularly between Day 9 and Day 1 (*p* < 0.05) and between Day 13 and Day 1 (*p* < 0.01), as shown in Figure 4. These results indicate that TMAO treatment significantly improved the endurance exercise capacity of the mice.

### 2.5. TMAO Enhanced Antioxidant Activity in the Mouse Gastrocnemius

Oxidative stress and lipid peroxide accumulation are widely recognized as key contributors to exercise-induced muscle fatigue. To investigate the correlation between TMAO treatment and its potential antioxidant activities in alleviating skeletal muscle fatigue, we quantified the levels of antioxidant compounds such as glutathione and taurine using NMR spectroscopy (Figure S3). In addition, we assessed the activity of the key antioxidant enzyme GSH-Px in the mouse gastrocnemius using a specialized assay kit.

**Figure 5:**
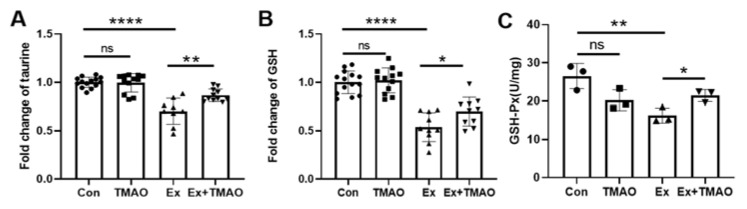
TMAO significantly increased the levels of taurine and glutathione, and the activity of GSH-Px, in the mouse gastrocnemius. (**A**) Fold change of taurine levels. (**B**) Fold change of glutathione levels. (**C**) Antioxidant activity of GSH-Px. Round, Con; Square, TMAO; Up Triangle, Ex; Down Triangle, Ex + TMAO. Triangle Statistical significance: ns *p* > 0.05, * *p* < 0.05; ** *p* < 0.01; **** *p* < 0.0001.

The Ex group showed significantly decreased levels of glutathione and taurine compared to the Con group (*p* < 0.0001). Significantly, TMAO treatment increased the levels of these two antioxidants (Figure 5A,B, Table S1). In addition, the Ex group also showed a decreased activity of GSH-Px compared to the Con group (*p* < 0.01) (Figure 5C). Similarly, TMAO treatment observably increased the activity of GSH-Px in the Ex + TMAO group compared to the Ex group (*p* < 0.05). Conversely, TMAO treatment did not result in statistically significant changes in the levels of glutathione and taurine nor the activity of GSH-Px in the TMAO group compared to the Con group.

### 2.6. TMAO Promoted the Expression of Nrf2 and Its Downstream Genes in the Mouse Gastrocnemius

**Figure 6:**
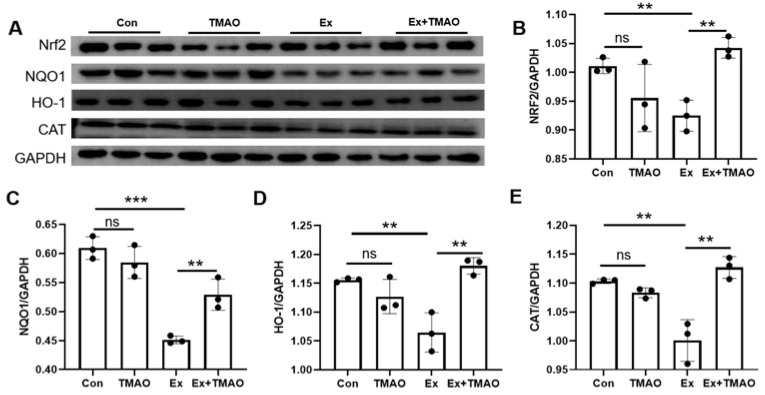
TMAO significantly increased the expression levels of Nrf2 and its downstream genes in the mouse gastrocnemius. (**A**) Western blotting analysis for the expression of Nrf2, NQO1, HO-1, and CAT. (**B**–**E**) Expression levels of Nrf2 (**B**), NQO1 (**C**), HO-1 (**D**), and CAT (**E**). Statistical significance: ns *p* > 0.05, ** *p* < 0.01; *** *p* < 0.001.

Given the central role of Nrf2 in modulating antioxidant enzymes and maintaining cellular redox balance, we analyzed proteins associated with the Nrf2 pathway. This comparative analysis revealed a significant decrease in the expression levels of Nrf2, NQO1, HO-1, and CAT proteins in the Ex group gastrocnemii compared to the Con group. Interestingly, TMAO treatment was able to partially restore the expression levels of these proteins in Ex + TMAO gastrocnemii (Figure 6). However, the expression levels of these proteins in the TMAO-treated gastrocnemii did not show significant differences compared to the Con group.

## 3. Discussion

Oxidative stress, a common byproduct of exercise, disrupts the balance between cellular pro- and antioxidant systems [23], potentially leading to cellular dysfunction [22]. The importance of endogenous antioxidants in reducing cellular damage during exercise highlights the importance of antioxidant supplementation [25,31]. In addition, the proliferation of muscle satellite cells plays a critical role in muscle development, repair during exercise, and adaptation to challenges such as disease, injury, and aging [32] and is essential for maintaining skeletal muscle homeostasis [33].

Our study demonstrates that TMAO treatment effectively protects myoblasts from oxidative stress-induced damage by upregulating the expression of Nrf2 and its downstream genes. We also observed that TMAO treatment significantly increased EST in mice, increased glutathione and taurine levels, improved GSH-Px activity, and stimulated the expression of Nrf2 and its related antioxidant genes in skeletal muscle.

Previous research has shown a significant decrease in urinary TMAO levels in elite athletes and active women following acute exercise [34], with an approximate 20–21% reduction in TMAO levels one hour after exercise and a gradual return to baseline [35]. Despite differing views, there is an emerging consensus regarding the decrease in TMAO levels following exercise. Additional studies have detected d9-TMAO and d9-trimethylamine in skeletal muscle six hours after ingestion of 50 mg d9-TMAO in healthy men [36], suggesting a possible role for TMAO during exercise.

Consistent with these findings, TMAO treatment significantly improved EST in mice, highlighting its role in reducing oxidative stress. This is consistent with the previously demonstrated potential of TMAO in various contexts, such as protecting against endoplasmic reticulum stress in diabetic rats [8] and effectively suppressing F2-isoprostane levels in MC65 cells [7]. TMAO also limits electron leakage from the mitochondrial electron transport chain, thereby reducing ROS production [37]. Our previous research supports these findings by demonstrating the efficacy of TMAO in alleviating H_2_O_2_-induced oxidative stress in C2C12 myoblasts [9]. Taken together, these results highlight the multiple antioxidant properties of TMAO and its potential as a therapeutic agent against oxidative stress-induced damage.

Nrf2, a critical transcription factor, is key to maintaining cellular redox balance and is primarily regulated by Keap1 [12,13]. Keap1 binds to the N-terminal regulatory domain of Nrf2 and represses its transcriptional activity [38]. Under oxidative stress, Nrf2 is released from Keap1 repression, translocates to the nucleus, binds to the antioxidant response element (ARE), and coordinates the transcription of several antioxidant genes, significantly enhancing cellular resistance to oxidative stress [39].

Our study, in line with the existing literature [40,41], focused on the critical role of Nrf2 and its downstream genes in combating oxidative stress. We observed a significant decrease in the expression of NQO1, HO-1, Nrf2, and CAT after H_2_O_2_ exposure in C2C12 myoblasts (Figure 2). Remarkably, TMAO treatment counteracted this decrease, suggesting that TMAO influences cellular oxidative stress responses through the Nrf2 pathway and its gene targets.

To confirm the essential role of Nrf2 in the protective mechanism of TMAO, we used siRNA to reduce Nrf2 expression. Knockdown of Nrf2 significantly attenuated the upregulation of Nrf2, NQO1, and HO-1 by TMAO (Figure 3), firmly establishing Nrf2 as a key upstream regulator in the protective effects of TMAO in C2C12 myoblasts under oxidative stress.

Skeletal muscle, which accounts for nearly half of total body mass [42], is critical for force generation through coordinated muscle fiber contractions during physical activity. This process is highly dependent on the precise regulation of the stress response and antioxidant defense mechanisms [43,44]. Due to its high oxygen consumption and metabolic activities, skeletal muscle and its satellite cells are continuously exposed to an oxidant-rich environment [45]. Intense exercise increases this oxidative load, potentially compromising muscle fiber integrity [46,47] and thus affecting muscle strength and overall health [42]. Therefore, the strategic use of antioxidants is critical to effectively prevent skeletal muscle damage.

Glutathione, a tripeptide composed of glutamate, cysteine, and glycine, derives its biological activity from the sulfhydryl (SH) group in cysteine [48,49]. As the predominant low-molecular-weight thiol in cells, glutathione is an important non-protein antioxidant that provides a robust defense against oxidative stress [48]. At the same time, taurine, a versatile free amino acid, plays an important role in calcium-dependent excitation–contraction processes and contributes to cell volume regulation and antioxidant defense in skeletal muscle [50], establishing it as a natural antioxidant.

Glutathione peroxidase (GSH-Px), a vital peroxidolytic enzyme, is critical in scavenging excess ROS and regulating redox balance by converting reduced glutathione to its oxidized form. It also converts toxic hydrogen peroxide to non-toxic hydroxyl compounds, thereby limiting peroxide-induced cell damage and preserving cell membrane integrity [51]. Our study showed a significant decrease in glutathione, taurine, and GSH-Px activity in the Ex mouse gastrocnemius, while the Ex + TMAO mouse gastrocnemius showed a significant upregulation of these antioxidant parameters (Figure 5), highlighting the role of TMAO in enhancing antioxidant defenses and mitigating exercise-induced oxidative stress in skeletal muscle.

Given the exercise-induced oxidative stress and the remarkable effect of TMAO treatment on EST prolongation in mice, we investigated oxidative stress markers in their skeletal muscles. Our results show a pronounced decrease in antioxidant levels (glutathione and taurine) and enzyme activity (GSH-Px), along with reduced expression of antioxidant proteins (Nrf2, NQO1, HO-1, and CAT) after exhaustive swimming. Notably, TMAO treatment resulted in significant increases in these antioxidants, enzymes, and proteins (Figure 5 and Figure 6), suggesting its efficacy in counteracting oxidative stress induced by exhaustive swimming through the Nrf2/antioxidant signaling pathway.

In conclusion, our research confirms the role of TMAO in reducing exercise-induced oxidative stress and improving muscle performance. This study lays the groundwork for further exploration of the long-term effects and applicability of TMAO supplementation in various exercise modalities. Our future research will aim to extend the duration of TMAO supplementation and examine its effects across a range of exercise intensities and types. By expanding the scope of our research, we aim to refine current knowledge and identify new strategies for optimizing TMAO supplementation, particularly in the context of exercise-induced oxidative stress and performance enhancement.

## 4. Materials and Methods

### 4.1. Reagents and Antibodies

C2C12 myoblasts were obtained from the Stem Cell Bank, Chinese Academy of Sciences, China (Shanghai, China). High purity hydrogen peroxide (H_2_O_2_) (30%, 10011218, China) was purchased from Sinopharm Chemical Reagent Co. Ltd. (Shanghai, China). TMAO of 95% purity was purchased from Sigma-Aldrich (St. Louis, MO, USA) (317594-5G). The GSH-Px assay kit (A005-1-2) was purchased from Nanjing Jiancheng Bioengineering Institute (Nanjing, China). Nrf2 small interfering RNA (siRNA) was provided by Santa Cruz Biotechnology, Inc. (sc-37049, Dallas, TX, USA). Lipofectamine 2000 (lip2000) for transfection was purchased from Thermo Fisher Scientific (68019, Waltham, MA, USA). Antibodies used in this study include Nrf2 (#12721, CST, USA), HO-1 (#43966, CST, USA), NQO1 (#62262, CST, USA), CAT (21260-1-AP, Proteintech, Wuhan, China), and GAPDH (10494-1-AP, Proteintech, China).

### 4.2. Cell Culture and Treatments

C2C12 cells were cultured in Dulbecco’s modified Eagle’s medium (DMEM; HyClone, Logan, UT, USA) supplemented with 10% fetal bovine serum (FBS; Gibco, Gaithersburg, MD, USA), 100 units/mL penicillin, and 100 μg/mL streptomycin in a 5% CO_2_ atmosphere at 37 °C.

For the experimental setup, C2C12 cells were divided into three groups: control (Con), H_2_O_2_-treated (H_2_O_2_), and H_2_O_2_-TMAO-treated (H_2_O_2_ + TMAO) (Figure S4). First, cells were grown in normal growth medium (GM) for 24 h until they reached 50–60% confluence. They were then exposed to 0.5 mM H_2_O_2_ for 2 h. After this exposure, the culture medium was changed to either normal GM for the H_2_O_2_-treated cells or normal GM supplemented with 5 mM TMAO for the H_2_O_2_-TMAO-treated cells. Both groups were further cultured for 24 h. At the same time, control cells were maintained in normal GM for the entire 50 h culture period.

### 4.3. Cell Transfection for Assessing the Impact of siRNA Nrf2

C2C12 cells were divided into six groups: untreated siControl (siC), H_2_O_2_-treated siControl (H_2_O_2_ siC), H_2_O_2_-TMAO-treated siControl (H_2_O_2_-TMAO siC), untreated siNrf2 (siN), H_2_O_2_-treated siNrf2 (H_2_O_2_ siN), and H_2_O_2_-TMAO-treated siNrf2 (H_2_O_2_-TMAO siN). Final concentrations of 0.5 mM for H_2_O_2_ and 5 mM for TMAO were added in the experimental setup (Figure S5).

C2C12 cells in the H_2_O_2_-treated and H_2_O_2_-TMAO-treated siC/siN groups (hereafter referred to as treated groups) were cultured in normal GM for 12 h until they reached 40–50% confluence. Then, either RNA (40 nM) or Lipofectamine 2000 reagent (32 nM) was diluted in Opti-MEM medium, and the diluted RNA was added to the diluted Lipofectamine 2000 reagent (1:1 ratio) and incubated for 5 min at room temperature. The RNA–lipid complex was added to the cells and incubated at 37 °C for 5 h. The Opti-MEM medium was then replaced with normal GM and the cells were exposed to H_2_O_2_ for 2 h. The culture medium was then switched, with H_2_O_2_-treated siC/siN cells receiving normal GM and H_2_O_2_-TMAO-treated siC/siN cells receiving normal GM supplemented with TMAO. All four treated groups were further cultured for 24 h. C2C12 cells in the untreated siC/siN group were maintained in GM for 26 h after transfection (5 h).

### 4.4. Cell Viability and Proliferation Assay

To explore the effects of H_2_O_2_ administration and TMAO supplementation on the proliferative ability of C2C12 cells, cell viability was assessed using the CellTiter 96 AQueous One Solution Cell Proliferation Assay Kit (Promega, Madison, WI, USA). C2C12 cells were seeded in 96-well plates at a density of 6 × 10^3^ cells per well and cultured as previously described [29]. Briefly, 20 μL of MTS (3-(4,5-dimethylthiazol-2-yl)-5-(3-carboxymethoxyphenyl)-2-(4-sulfophenyl)2H-tetrazolium) was added to each well, and the plates were covered with tin–platinum paper before incubation in the dark at 37 °C for 3 h. Formazan absorbance was then measured at 490 nm using a microplate reader (BioTek, Winooski, VT, USA).

To measure the proliferative effect of TMAO supplementation on the H_2_O_2_-impaired C2C12 cells, the automated cell counting method was used. First, the adherent C2C12 cells were washed three times with PBS to remove dead cells. Then, 1 mL of trypsin was added to the cell culture dish for digestion for 1 min, followed by the addition of 1 mL of DMEM to create a cell suspension. This suspension was then transferred to a 2 mL Eppendorf tube and centrifuged at 1000 rpm for 30 s in a microcentrifuge. After removing the supernatant, the cells were resuspended in 1 mL of PBS. After 10-fold dilution, 20 μL of the cell suspension was added to a counting plate (CO010101, Countstar, Shanghai, China). The plate was placed in an automated cell counter (IC1000, Countstar, Shanghai, China) and the average was calculated.

### 4.5. ROS Level

Intracellular ROS levels were measured with the fluorescent cell permeable probe H_2_DCFDA (2′,7′-dichlorofluorescin diacetate) (Sigma, D6883). C2C12 myoblasts were cultured in DMEM as described above, then washed three times with PBS, followed by incubation with H_2_DCFDA (5 μM) for 30 min at 37 °C. Subsequently, the cells were washed again with PBS three times. Intracellular ROS levels were determined by the fluorescence intensity of H_2_DCFDA detected by a fluorescence microplate reader.

### 4.6. Animals and Ethical Approval

All experimental protocols were approved by the Animal Care and Use Committee of Beijing Sport University (2021127A). The study strictly adhered to the relevant institutional and governmental guidelines and regulations governing the ethical treatment of animals. Male C57BL/6 mice, aged 11 weeks, were obtained from Beijing Huafukang Biotechnology Co., Ltd. (Beijing, China). These mice were carefully housed in a controlled environment with a 12 h light/12 h dark cycle to promote their overall well-being and reduce stress.

### 4.7. Western Blotting

Protein isolation from 50 mg of gastrocnemius tissue was conducted utilizing RIPA protein extraction reagents (Thermo Fisher, Waltham, MA, USA). Protein concentrations were determined using the BCA protein analysis kit (Beyotime, Shanghai, China). An equal amount of protein from each sample was then subjected to SDS-PAGE and transferred to PVDF membranes (GE, Freiburg, Germany). Following a 1 h blocking step with 5% nonfat dry milk, the membranes were probed with anti-Nrf2, NQO1, HO-1, CAT, and GAPDH antibodies overnight at 4 °C. After thorough washing with TBST, the membranes were incubated with horseradish peroxidase-conjugated antibodies, and protein bands were visualized by enhanced chemiluminescence. The density of protein bands was quantified using ImageJ software 1.8.0 (National Institutes of Health, Bethesda, Rockville, MD, USA).

### 4.8. Animal Experimental Design

**Figure 7:**
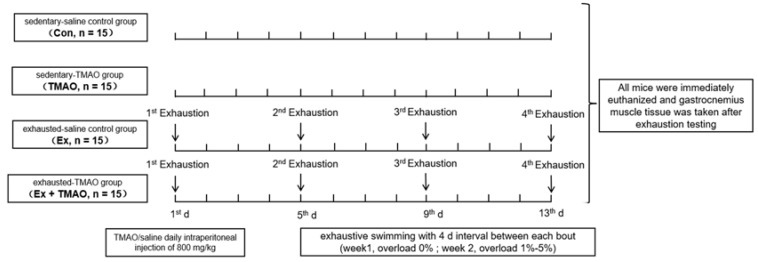
Schematic representation of the animal experimental design.

The experimental design is shown in Figure 7. Mice were randomly assigned to one of four groups: the sedentary saline control group (Con), the sedentary TMAO-treated group (TMAO), the exhausted saline control group (Ex), and the exhausted TMAO-treated group (Ex + TMAO).

To ensure effective adaptation for the swimming tests, we implemented a detailed two-week adaptation protocol in a water tank measuring 80 cm long, 50 cm wide, and 90 cm deep. Mice underwent training sessions five days per week to adapt to the aquatic environment, which was maintained at a constant temperature of 30 ± 2 °C. As outlined in Table 1, the adaptive swimming program included a variety of swimming activities tailored to gradually introduce the mice to swimming. Consistent with protocols from previous studies [52], mice in the exhausted group wore additional weights ranging from 1% to 5% of their body weight attached to their tails with lead skin from the first to the fifth day of the second week. Buoyancy control measures were consistently applied during the swimming sessions. During the first week, exercise duration was gradually increased from 10 to 50 min over five days. During the second week, a uniform daily swimming regimen of 30 min was followed (see Table 1). The exhausted groups underwent four exhaustion trials with a four-day rest period between each trial. A mouse was considered exhausted if it could not keep its head above water for more than 10 s. After the swimming exercise, each mouse was gently dried with a towel and hair dryer before being returned to its cage. Before the final exhaustion test (the 4th exhaustion), the mice were fasted for 4 h and then immediately subjected to the swimming test. After the swimming exercise, the epidermis was carefully removed, and bilateral gastrocnemius tissues were rapidly excised, snap frozen in liquid nitrogen, and then stored at −80 °C for future biochemical analysis.

### 4.9. Preparation of NMR Samples

Approximately 70 mg of gastrocnemius tissue was obtained after excision of the Achilles tendon and superficial fascia. A solvent mixture consisting of methanol, chloroform, and water in a 4:4:2.85 ratio was used to isolate aqueous metabolites from the skeletal muscle. The tissue was then homogenized at a frequency of 65 Hz for 60 s. This was followed by two minutes of vortexing and 15 min of centrifugation at 12,000 rpm and 4 °C. The upper aqueous layer was then dried with a stream of nitrogen and lyophilized to a fine powder. This powder was resuspended in 550 µL of NMR buffer to achieve a concentration of 1 mM sodium 3-(trimethylsilyl) propionate-2,3,3,4 (TSP). All samples were thoroughly vortexed and centrifuged before being transferred to 5 mm NMR tubes to prepare them for subsequent NMR spectroscopic analysis.

### 4.10. NMR Measurements

One-dimensional (1D) ^1^H-NMR spectra were acquired at 25 °C on a Bruker Avance III 850 MHz NMR spectrometer (Bruker Bio Spin, Ettlingen, Germany) equipped with a TCI cryoprobe. The NOESYGPPR1D pulse sequence was used with a relaxation delay of 4 s, 32 scans, and a spectral width of 20 ppm. The acquired spectra were then processed using MestReNova 9.0 software (Mestrelab Research S.L., Santiago de Compostela, Spain), including phase correction and baseline correction. The methyl groups of the TSP molecule were set to 0 ppm for chemical shift calibration. The spectral region from 9.5 to 0.6 ppm was segmented into 0.001 ppm bins for further statistical analysis. The water region from 5.2 to 4.7 ppm was excluded to eliminate distortion from the residual water resonance in all 1D ^1^H spectra.

Singlets or non-overlapping resonances in each NMR spectrum were selected to calculate metabolite integrals, normalized using both the TSP spectral integral and the tissue weight. These normalized integrals represented the relative levels of the assigned metabolites, presented as mean ± standard deviation (SD). Pairwise comparisons of the relative levels of metabolites were performed using GraphPad Prism software (version 8.3.0, La Jolla, CA, USA).

### 4.11. Measurement of GSH-Px Activity in the Mouse Gastrocnemius

Gastrocnemius tissues were processed according to the protocol described in the instructions for the GSH-Px assay kit. Briefly, the tissues were mixed with the assay reagents and incubated at room temperature for 15 min. The absorbance value of the mouse gastrocnemius tissue was then measured at 412 nm using a multimode microplate reader (POLAR 4star Omega, Offenburg, Germany) with a 1 cm optical path, according to the manufacturer’s instructions. The GSH-Px activity was then calculated from the measured absorbance value.

### 4.12. Statistical Analysis

Statistical analyses were performed using GraphPad Prism software version 8.3.0 (La Jolla, CA, USA). Data are expressed as mean ± standard deviation (SD). Pairwise comparisons were performed using a two-tailed Student’s *t*-test. Differences with *p* < 0.05 were considered statistically significant.

## 5. Conclusions

This study represents a significant advance in understanding the benefits of TMAO treatment in mitigating exercise-induced oxidative stress, particularly through its intricate interaction with the Nrf2 signaling pathway. Our findings elucidate the molecular mechanisms of TMAO treatment, paving the way for its potential application as an effective sports nutrition supplement. The knowledge gained from this research provides a solid foundation for future efforts to optimize muscle performance and reduce oxidative damage through strategic nutritional approaches.

## Figures and Tables

**Table 1:** The adaptive swimming exercise program.

Date	Training Time (min)	Overload (%)
Week 1		
Day 1	10	no
Day 2	20	no
Day 3	30	no
Day 4	40	no
Day 5	50	no
Week 2		
Day 1	30	1
Day 2	30	2
Day 3	30	3
Day 4	30	4
Day 5	30	5

## Data Availability

The data are contained within the article or Supplementary Material.

## Supplementary Materials

The following supporting information can be downloaded at: https://www.mdpi.com/article/10.3390/molecules29040759/s1. Figure S1: The chemical structure of TMAO; Figure S2: Effects of H_2_O_2_ administration and TMAO supplementation on the proliferative ability of C2C12 cells. Figure S3: Local amplified regions of glutathione and taurine peaks in a typical 850 MHz ^1^H-NMR spectrum recorded on aqueous extracts derived from mouse gastrocnemius. Figure S4: Schematic representation of the experimental design for C2C12 cells in growth medium (GM) supplemented with either H_2_O_2_ or H_2_O_2_ + TMAO. Figure S5: Schematic representation of the transfection experiment for C2C12 cells in growth medium supplemented with either H_2_O_2_ or H_2_O_2_ + TMAO. Table S1: Metabolite (glutathione and taurine) levels in the Con, TMAO, Ex and Ex + TMAO groups of mouse gastrocnemius based on relative NMR integrals.
