# Supplementary material for: Trimethylamine N-Oxide Improves Exercise Performance by Reducing Oxidative Stress through Activation of the Nrf2 Signaling Pathway
Source: Molecules. 2024 Feb 6;29(4):759. doi: 10.3390/molecules29040759 (PMC10893042; doi:10.3390/molecules29040759)
Supplement: Supplementary file 1 [file molecules-29-00759-s001.zip › supplementary.htm]

Supplementary Materials

Figure S1.

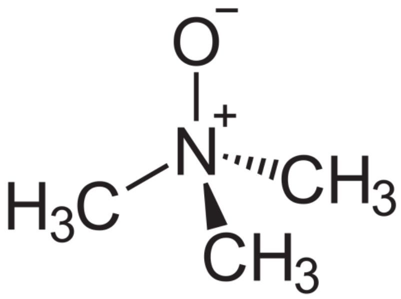

Figure S1. The chemical structure of TMAO

Figure S2.

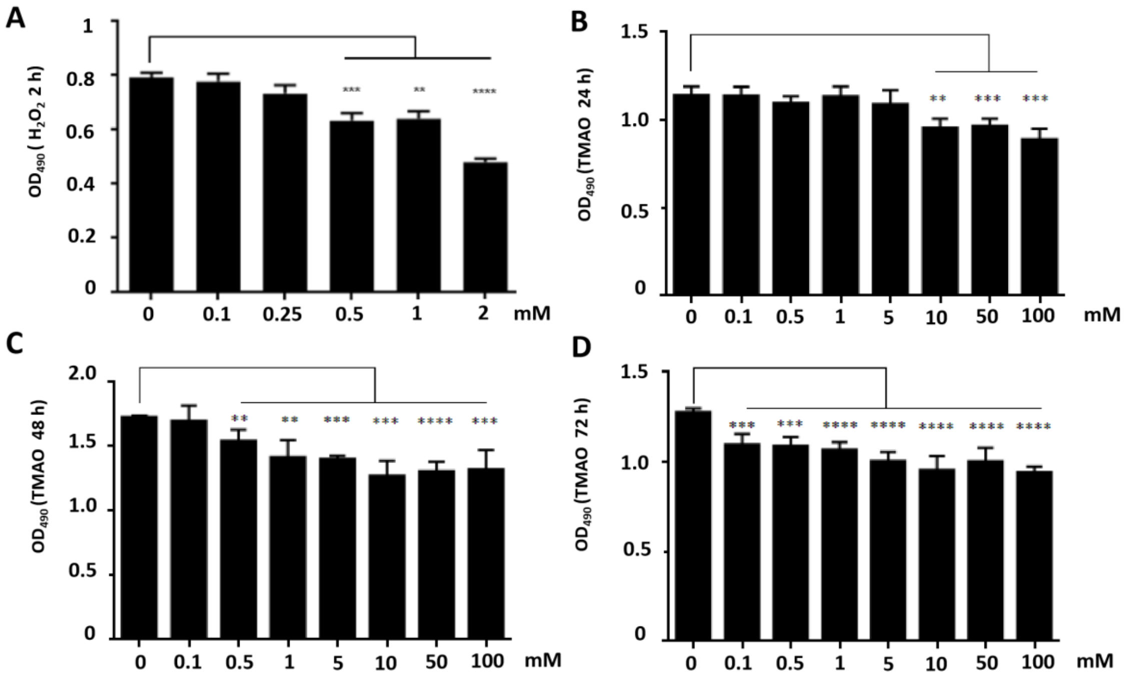

Figure S2. Effects of H_2O_2 administration and TMAO supplementation on the proliferative ability of C2C12 cells. (A) Cell viability of cells treated with different concentrations of H_2O_2; (B-D) Cell viability of cells treated with different concentrations of TMAO for different time periods: 24 h (B), 48 h (C), and 72 h (D). Cell viability, as assessed by the MTS assay, is represented by $OD_{490\ nm}$, with each

condition tested in six replicates. Statistical significance: **, $p < 0.01$; ***, $p < 0.001$; ****, $p < 0.0001$.

Figure S3.

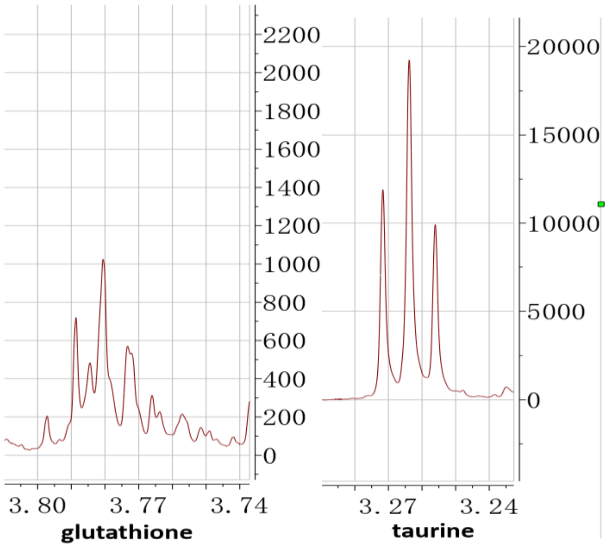

Figure S3. Local amplified regions of glutathione and taurine peaks in a typical 850 MHz ^{1}H-NMR spectrum recorded on aqueous extracts derived from mouse gastrocnemius. The TSP peak was used as the chemical shift reference (δ 0.00).

Figure S4.

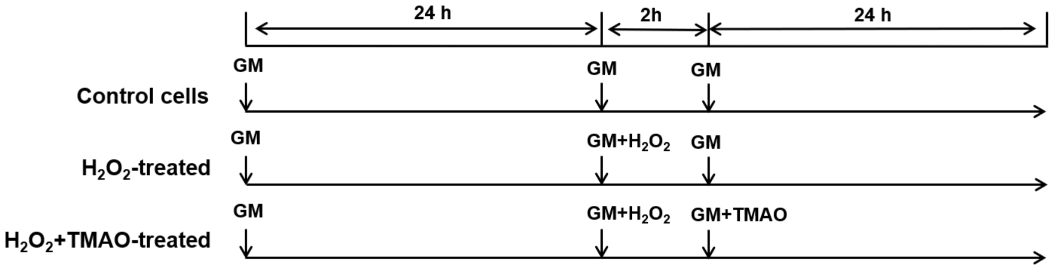

Figure S4. Schematic representation of the experimental design for C2C12 cells in growth medium (GM) supplemented with either H_2O_2 or H_2O_2 + TMAO.

Figure S5.

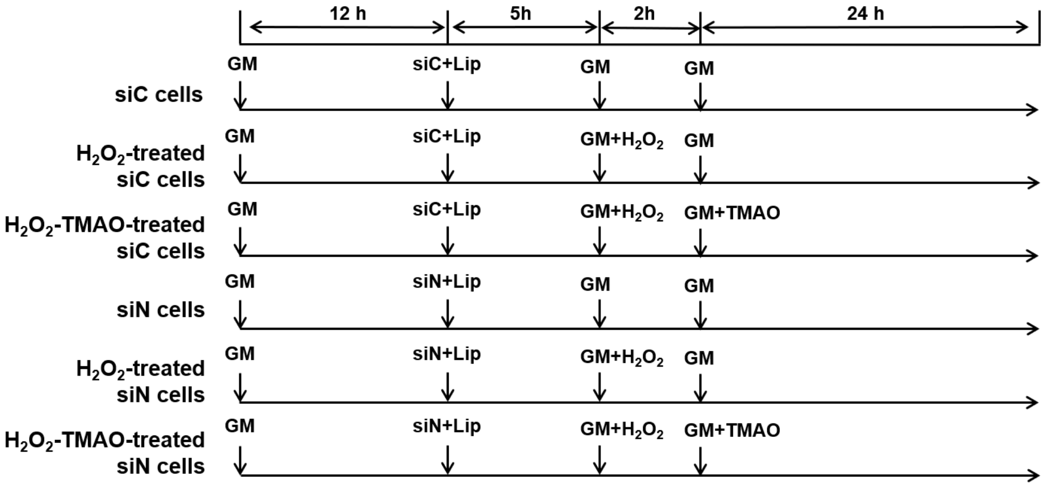

Figure S5. Schematic representation of the transfection experiment for C2C12 cells in growth medium supplemented with either H_2O_2 or H_2O_2 + TMAO. Abbreviations: GM, growth medium; siC, siControl; siN, siRNA Nrf2; Lipofectamine 2000, Lip; hour, h.

Table S1. Metabolite (glutathione and taurine) levels in the Con, TMAO, Ex and Ex+TMAO groups of mouse *g*astrocnemius based on relative NMR integrals.

Metabolites	Con	TMAO	Ex	Ex+TMAO
glutathione	0.21±0.02	0.21±0.03	0.11±0.03****	0.15±0.03$^{\#}$
taurine	1.72±0.09	1.72±0.16	1.21±0.25****	1.49±0.11$^{\#\#}$

Statistical significance: *, Ex *vs*. Con; ****, $p < 0.0001$; # TMAO+Ex *vs*. Ex; #, $p < 0.05$; ##, $p < 0.01$.
